# Supplementary material for: CLASP2 promotes repair of kinesin-1 damage to the microtubule lattice
Source: bioRxiv. 2026 Jun 30:2026.06.29.735199. Preprint. [Version 1] doi: 10.64898/2026.06.29.735199 (PMC13345139; doi:10.64898/2026.06.29.735199)
Supplement: Supplement 1 [file NIHPP2026.06.29.735199v1-supplement-1.pdf]

bioRxiv preprint doi: https://doi.org/10.64898/2026.06.29.735199; this version posted June 30, 2026. The copyright holder for this preprint (which was not certified by peer review) is the author/funder, who has granted bioRxiv a license to display the preprint in perpetuity. It is made available under a CC-BY 4.0 International license.

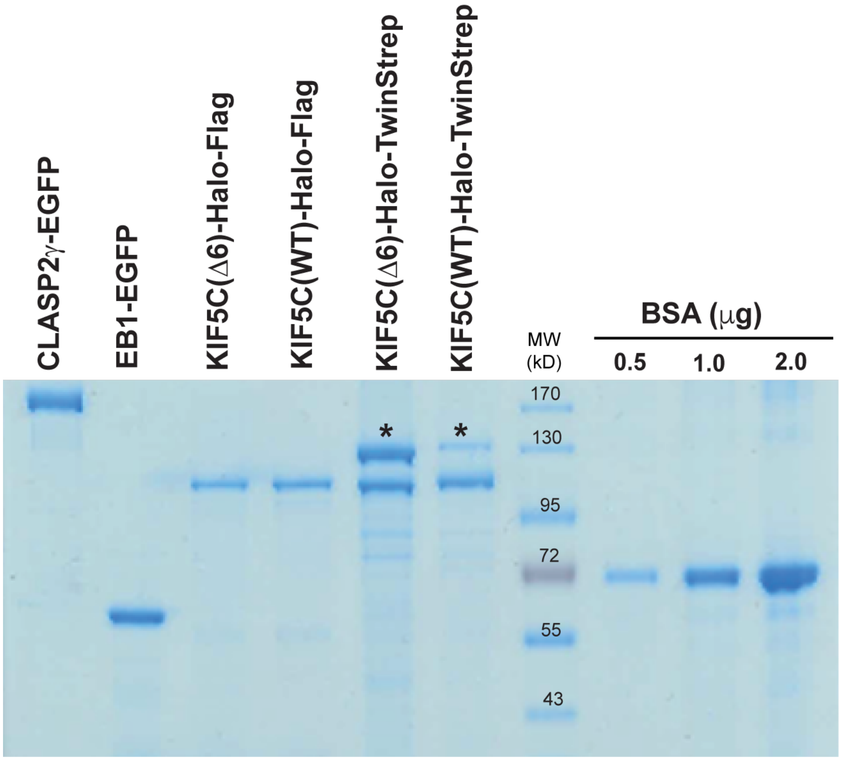

Fig. S1. SDS-PAGE of purified proteins. Coomassie-stained gel of purified proteins used in this work and BSA standards. Asterisks indicate protein that co-purifies with KIF5C-Halo-TwinStrep and identified as pyruvate carboxylase by mass spectrometry. MW: molecular weight markers in kD.

bioRxiv preprint doi: https://doi.org/10.64898/2026.06.29.735199; this version posted June 30, 2026. The copyright holder for this preprint (which was not certified by peer review) is the author/funder, who has granted bioRxiv a license to display the preprint in perpetuity. It is made available under a CC-BY 4.0 International license.

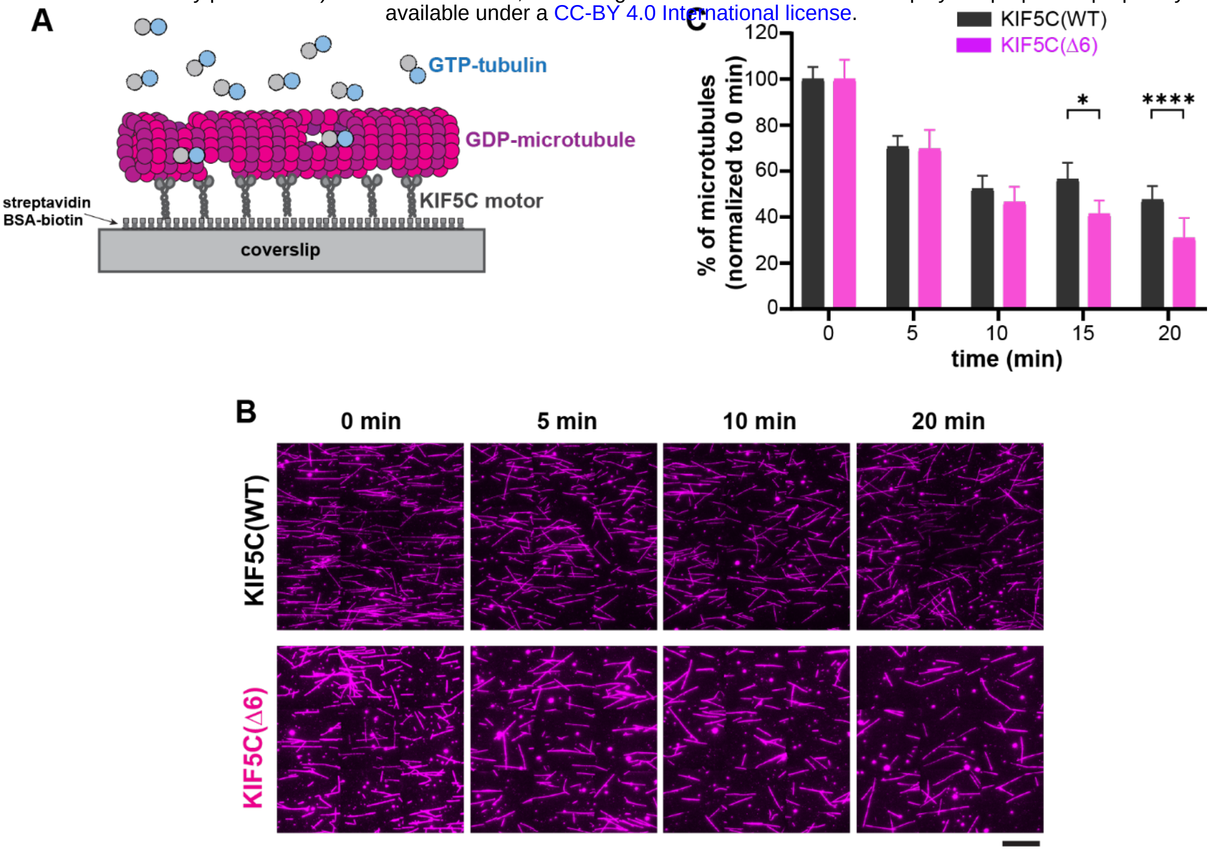

Fig S2. Microtubule destruction assay controls. (A) Schematic of microtubule destruction assay. KIF5C(WT) or KIF5C(Δ6) motors were attached to coverslips and then glycerol-stabilized GDP-microtubules were added in the presence of 2 mM ATP, 1 mM GTP and 7 μM unlabeled free tubulin. (B) Representative images of GDP-microtubules at 0, 5, 10, and 20 min after gliding driven by KIF5C(WT) or KIF5C(Δ6) motors. Scale bar: 20 μm. (C) Quantification of microtubule destruction over time by (black) KIF5C(WT) or (magenta) KIF5C(Δ6). The total number of microtubules was counted per field of view at 0 min (immediately after ATP addition to start KIF5C motility) and then after 5, 10, 15, or 20 min. The percent of microtubules remaining in each FOV was determined, averaged for 27 FOV across three independent experiments, and normalized to the 0 min time point (error bars: SE). * $p<0.05$, **** $p<0.0001$ (two-tailed t test).

bioRxiv preprint doi: https://doi.org/10.64898/2026.06.29.735199; this version posted June 30, 2026. The copyright holder for this preprint (which was not certified by peer review) is the author/funder, who has granted bioRxiv a license to display the preprint in perpetuity. It is made available under a CC-BY 4.0 International license.

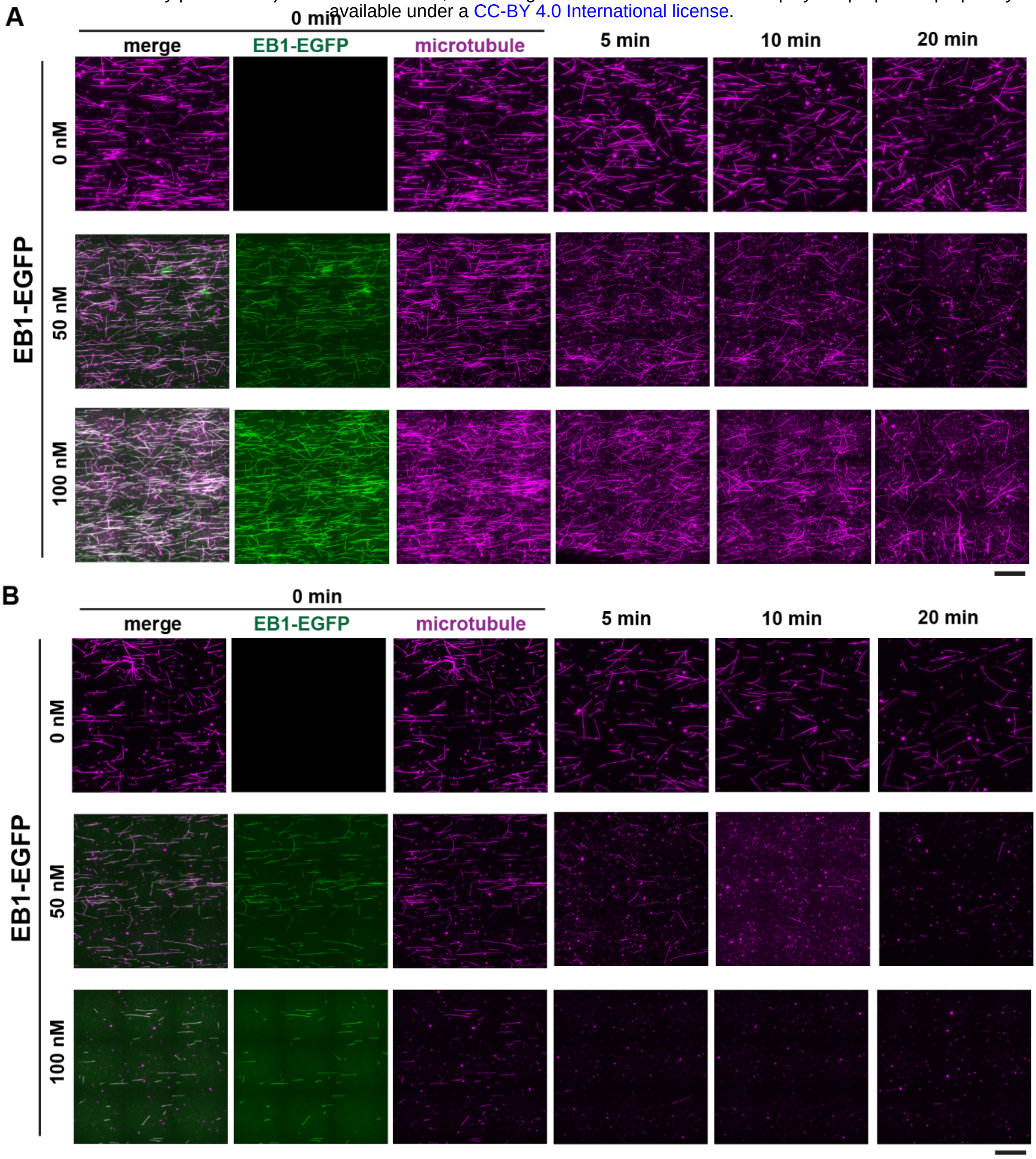

Fig S3. EB1 does not protect microtubules from kinesin-induced destruction. Representative images of microtubules remaining after damage induced by (A) KIF5C(WT) or (B) KIF5C(Δ6) in the absence or presence of the indicated concentrations of EB1-EGFP. Scale bar: 20 μm.

bioRxiv preprint doi: https://doi.org/10.64898/2026.06.29.735199; this version posted June 30, 2026. The copyright holder for this preprint (which was not certified by peer review) is the author/funder, who has granted bioRxiv a license to display the preprint in perpetuity. It is made available under a CC-BY 4.0 International license.

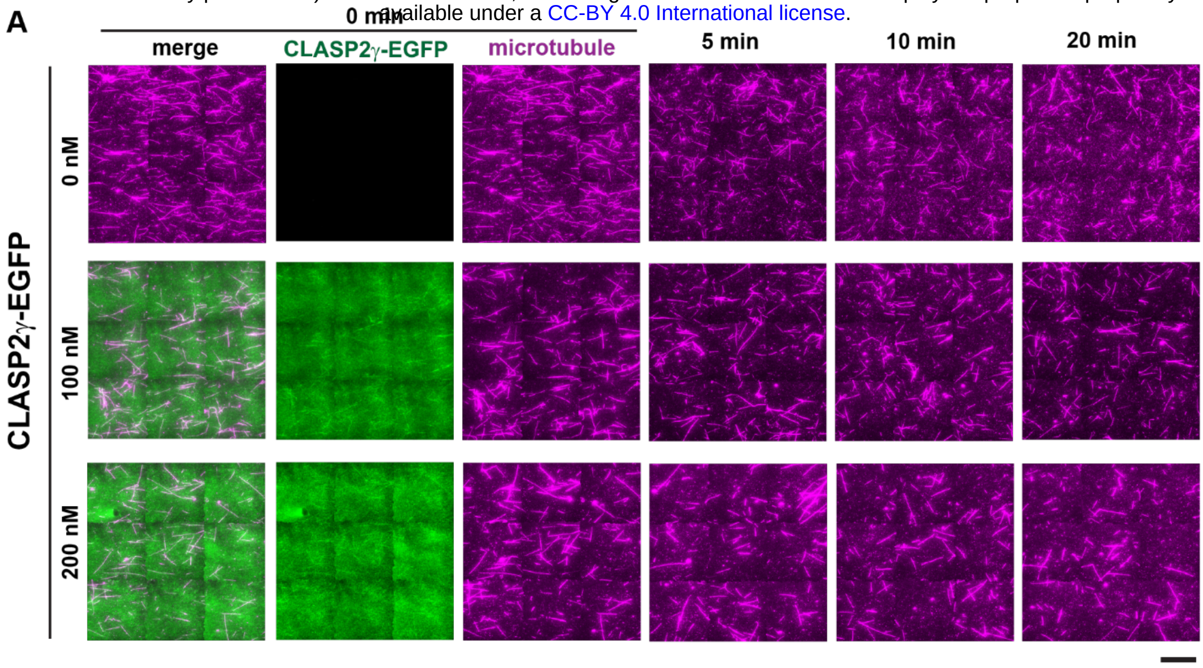

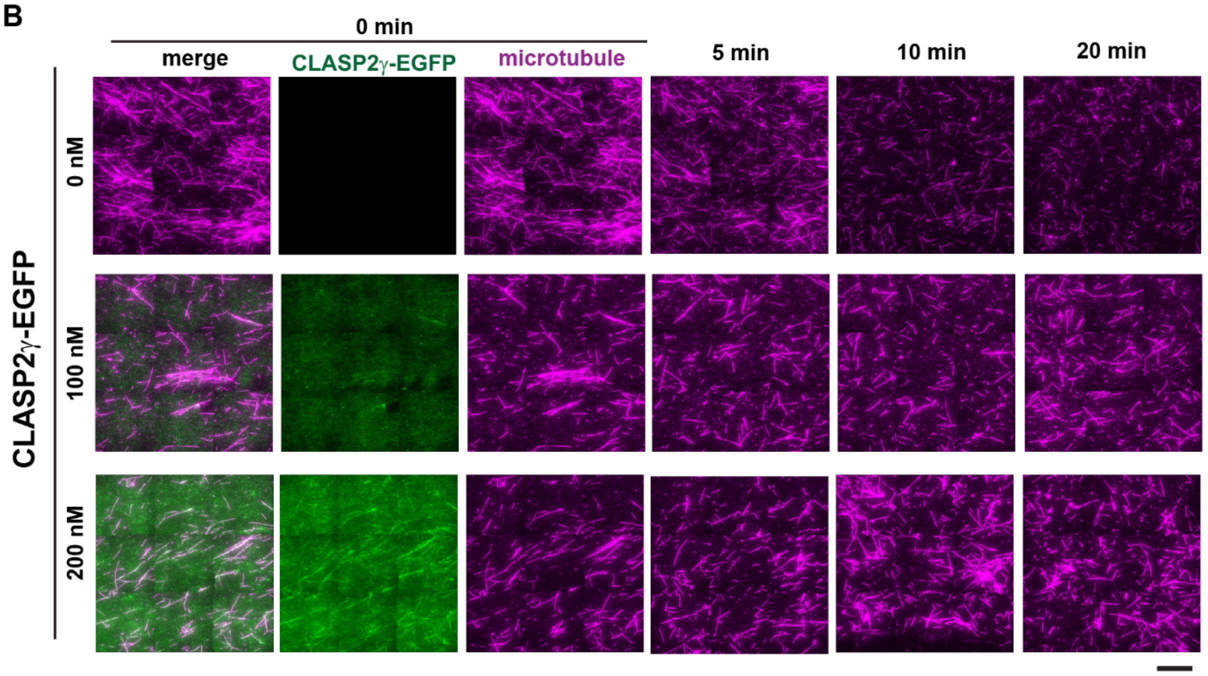

Fig S4. CLASP2γ protects microtubules from kinesin-induced destruction. Representative images of microtubules remaining after damage induced by (A) KIF5C(WT) or (B) KIF5C(Δ6) in the absence or presence of the indicated concentrations of CLASP2γ-EGFP. Scale bar: 20 μm.

bioRxiv preprint doi: https://doi.org/10.64898/2026.06.29.735199; this version posted June 30, 2026. The copyright holder for this preprint (which was not certified by peer review) is the author/funder, who has granted bioRxiv a license to display the preprint in perpetuity. It is made available under a CC-BY 4.0 International license.

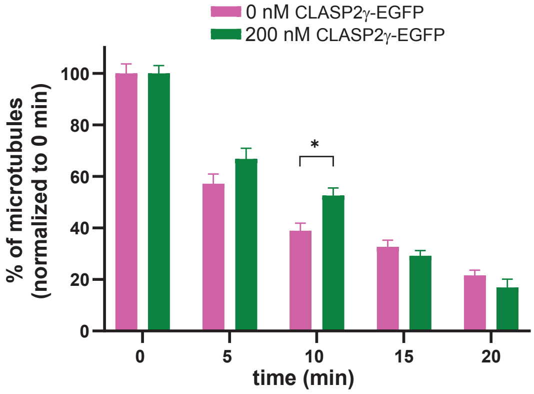

Fig S5. CLASP2γ does not protect microtubules from kinesin-induced damage in absence of free tubulin. Quantification of the microtubules remaining after KIF5C(Δ6)-induced microtubule damage over time in absence (magenta) or presence of 200 nM CLASP2γ-EGFP (green). The total number of microtubules was counted per field of view at 0 min (immediately after ATP addition to start KIF5C motility) and then after 5, 10, 15, or 20 min. The percent of microtubules remaining in each FOV was determined, averaged for 27 FOV across three independent experiments, and normalized to the 0 min time point (error bars: SE). * $p<0.05$ (two-tailed t test).

bioRxiv preprint doi: https://doi.org/10.64898/2026.06.29.735199; this version posted June 30, 2026. The copyright holder for this preprint (which was not certified by peer review) is the author/funder, who has granted bioRxiv a license to display the preprint in perpetuity. It is made available under a CC-BY 4.0 International license.

Movie legends.

Movie S1: KIF5C(WT)-induced microtubule destruction. Representative movie of microtubule destruction assayswith glycerol-stabilized GDP-microtubules driven by ~200 nM surface-bound KIF5C(WT) motors in the presence of 1 mM GTP, 2 mM ATP, and 7 μM free tubulin. Images were acquired for 5 min at 5 sec intervals.

Movie S2: KIF5C(Δ6)-induced microtubule destruction. Representative movie of microtubule destruction assay with glycerol-stabilized GDP-microtubules driven by ~200 nM KIF5C(Δ6) motors in presence of 1 mM GTP, 2 mM ATP, and 7 μM free tubulin. Images were acquired for 5 min at 5 sec intervals.
